# Supplementary material for: MetaRibo-Seq measures translation in microbiomes
Source: Nat Commun. 2020 Jun 29;11:3268. doi: 10.1038/s41467-020-17081-z (PMC7324362; doi:10.1038/s41467-020-17081-z)
Supplement: Supplementary file 10 — Supplementary Data 7 [file 41467_2020_17081_MOESM10_ESM.zip › File2/Confidence_High_Taxonomy/330875_out.krona.html]

Javascript must be enabled to view this page.

members
magnitude
magnitudeUnassigned
count
unassigned
taxon
rank

330875\_out

2

2
2
superkingdom

phylum
32066
1

1
203490
class

203491
order
1

1
1129771
family

1
genus
32067

1

SRS101439\_contig\_number\_35040
157687
species

201174
phylum
1

1760
class
1

1
2037
order

family
2049
1

1654
genus
1

272548
species
1

SRS143885\_contig\_number\_3288
